# MicroRNA-146a-5p-modified human umbilical cord mesenchymal stem cells enhance protection against diabetic nephropathy in rats through facilitating M2 macrophage polarization

**DOI:** 10.1186/s13287-022-02855-7

**Published:** 2022-04-27

**Authors:** Yaqi Zhang, Xi Le, Shuo Zheng, Ke Zhang, Jing He, Mengting Liu, Chengshu Tu, Wei Rao, Hongyuan Du, Yu Ouyang, Changyong Li, Dongcheng Wu

**Affiliations:** 1 Department of Biochemistry and Molecular Biology, Wuhan University School of Basic Medical Sciences, Wuhan, China; 2 Wuhan Hamilton Biotechnology Co., Ltd., Wuhan, China; 3 Department of Physiology, Wuhan University School of Basic Medical Sciences, Wuhan, China; 4 Guangzhou Hamilton Biotechnology Co., Ltd, Wuhan, China

**Keywords:** Mesenchymal stem cells, Diabetic nephropathy, MicroRNAs, Macrophage polarization

## Abstract

**Background:**

Diabetic nephropathy (DN) is a severe complication of diabetes mellitus and a common cause of end-stage renal disease (ESRD). Mesenchymal stem cells (MSCs) possess potent anti-inflammatory and immunomodulatory properties, which render them an attractive therapeutic tool for tissue damage and inflammation.

**Methods:**

This study was designed to determine the protective effects and underlying mechanisms of human umbilical cord-derived MSCs (UC-MSCs) on streptozotocin-induced DN. Renal function and histological staining were used to evaluate kidney damage. RNA high-throughput sequencing on rat kidney and UCMSC-derived exosomes was used to identify the critical miRNAs. Co-cultivation of macrophage cell lines and UC-MSCs-derived conditional medium were used to assess the involvement of macrophage polarization signaling.

**Results:**

UC-MSC administration significantly improved renal function, reduced the local and systemic inflammatory cytokine levels, and attenuated inflammatory cell infiltration into the kidney tissue in DN rats. Moreover, UC-MSCs shifted macrophage polarization from a pro-inflammatory M1 to an anti-inflammatory M2 phenotype. Mechanistically, miR-146a-5p was significantly downregulated and negatively correlated with renal injury in DN rats as determined through high-throughput RNA sequencing. Importantly, UC-MSCs-derived miR-146a-5p promoted M2 macrophage polarization by inhibiting tumor necrosis factor receptor-associated factor-6 (TRAF6)/signal transducer and activator of transcription (STAT1) signaling pathway. Furthermore, miR-146a-5p modification in UC-MSCs enhanced the efficacy of anti-inflammation and renal function improvement.

**Conclusions:**

Collectively, our findings demonstrate that UC-MSCs-derived miR-146a-5p have the potential to restore renal function in DN rats through facilitating M2 macrophage polarization by targeting TRAF6. This would pave the way for the use of miRNA-modified cell therapy for kidney diseases.

**Supplementary Information:**

The online version contains supplementary material available at 10.1186/s13287-022-02855-7.

## Introduction

Diabetic nephropathy (DN) is one of devastating microvascular complication of diabetes mellitus and the most common cause of end-stage renal disease (ESRD), with 30–40% mortality [1–3]. DN is characterized by specific renal structure and functional alterations such as glomerular hyperfiltration, microalbuminuria, thickening of the glomerular basement membrane, interstitial fibrosis, and hypertrophy of mesangial cells [4, 5]. Despite current pharmacological treatments, including strategies for optimizing glycemic control and inhibitors of the renin-angiotensin system, these conventional treatments provide incomplete kidney protection [6]. Hence, there is an urgent need for novel therapeutic approaches that efficiently delay the disease progression.

The initiating mechanisms underlying the development and progression of renal injury in DN are not well understood, but current knowledge indicates that its pathogenesis is multifactorial. Compelling and increasing evidence has clearly demonstrated that immunity and inflammation play a paramount role in the pathogenesis of DN. Indeed, DN is associated with both systemic and local renal inflammation with the participation of crucial inflammatory cells such as macrophages [7–11]. In response to various signals, macrophages may undergo classical M1 activation or alternative M2 activation. The M1 phenotype is characterized by the expression of high levels of proinflammatory cytokines, high production of oxygen intermediates, and promotion of Th1 response. In contrast, M2 macrophages are considered to be involved in immunomodulation and promotion of tissue remodeling [12, 13].

With the advent of cell therapy, mesenchymal stem cells (MSCs) are considered as the most attractive cell source for regenerative medicine and provide a promising strategy to against DN. MSCs are self-renewing and multipotent progenitors that can differentiate into a variety of cell types [14]. MSCs can be obtained from various tissues, including bone marrow, skeletal muscle, dental pulp, adipose tissue and umbilical cord [15–17]. Growing evidence in recent years has revealed that MSCs have multiple biological functions, including immunomodulation, anti-inflammation, anti-apoptosis and anti-fibrosis [18]. Because of these characteristics, MSCs have been applied in various diseases, such as respiratory diseases [19], circulatory system diseases [20], nervous system diseases [21], and kidney diseases [22–24]. However, the underlying mechanisms of these beneficial effects are not completely elucidated. Of note, human umbilical cord-derived MSCs (UC-MSCs) are much younger and lower immunogenic, and have a higher yield without ethical issues and invasive procedures. Importantly, UC-MSCs can secrete a wide range of multifunctional factors. Thus human UC-MSCs are considered to be a better choice for clinical applications compared to many other MSCs [25].

MicroRNAs (miRNAs) are a class of noncoding single-stranded small RNA with 20–22 nucleotides in length [26]. miRNAs exert a regulatory effect on a wide range of biological cell processes including cell apoptosis, proliferation, and inflammation by incompletely pairing with the 3′-untranslated region of the target mRNA [27]. As an endogenous RNA, miRNA maintains stable and conservative in mammals. It has been estimated there more than 2500 mature miRNAs in human genome could regulate expression of gene in physiology and disease [28, 29]. Previous studies have implicated that MSCs-derived exosomes mediate intercellular communications through exchange of proteins, mRNAs, and mostly the miRNAs, which negatively regulate expression of target genes in diverse biological processes [30, 31]. However, it remains unclear whether and how UC-MSCs-derived miRNA may regulate macrophage polarization in the pathogenesis of DN.

In this study, using streptozotocin (STZ)-induced DN rat model and in vitro co-culture experiments, we investigated the efficacy and mechanism of UC-MSCs-based protection against renal injury in DN. Our findings demonstrate that miR-146a-5p mediates the beneficial effects of UC-MSCs on functional recovery in DN through facilitating M2 macrophage polarization by targeting TRAF6-STAT1 signaling pathway.

## Materials and methods

### Animal experiments

Eight-week-old adult male Sprague Dawley rats (200–220 g) were obtained from Hubei provincial center for disease control and prevention (Wuhan, China). This study was carried out following the recommendations in the guide for Institutional Animal Care and Use Committee procedures of Hubei Provincial Center for Food and Drug Safety Evaluation (Permit Number: 202020005, 202020108). After one-week adaptive feeding, rats were rendered diabetic were induced by a single intraperitoneal (I.P) injection of STZ (S0130, Sigma, USA), in a dosage of 60 mg/kg dissolved in 0.1 M cold citrate buffer (pH 4.5) [32, 33]. After 24 h, rats were checked for blood glucose, and those with blood glucose more than 16.7 mM for three consecutive days were confirmed as diabetes. Metabolic cages were used to collect urine from diabetes rats for measuring volume and protein concentration after STZ treatment on 4–6 weeks, and proteinuria ≥ 30 mg/24 h was verified as DN. At 12 weeks after STZ or citrate buffer injection, rats were euthanized and blood and urine were collected for renal function detection. Serum creatinine and serum urea nitrogen ware measured with kit according to the manufacturer's instructions (C011-1-1, C013-1-1, Nanjing Jiancheng Bioengineering Institute, China). BCA kit (P0012S, Beyotime, China) was used to measure the concentration of the protein in urine.

### Experimental design and treatment protocol

Rats were randomly divided into the following groups: (1) normal control group (*n* = 5); (2) DN group (*n* = 6), (3) UC-MSC treatment group I (*n* = 8): DN rats were treated with two injections of 2 × 10^6^ UC-MSCs through the tail vein at week 7 and 8; (4) UC-MSC treatment group II (*n* = 8): DN rats were treated with two injections of 2 × 10^6^ UC-MSCs through the tail vein at week 9 and 10.

In order to explore the therapeutic effect of miR-146a-5p-modified UC-MSCs on DN, we performed another animal experiment and the UC-MSCs were injected at week 9 and 10. Rats were randomly divided into the following groups: (1) normal control group (*n* = 4); (2) normal control plus miR-146a-5p mimic-transfected UC-MSC treatment group (*n* = 4); (3) normal control plus miR-146a-5p mimic NC-transfected UC-MSC treatment group (*n* = 4); (4) DN group (*n* = 4); (5) DN plus miR-146a-5p mimic-transfected UC-MSC treatment group (*n* = 4); (6) DN plus miR-146a-5p mimic NC-transfected UC-MSC treatment group (*n* = 4).

#### Enzyme-linked immunosorbent assay

The levels of IL-1β and IL-10 in the kidney tissues and serum from experimental rats were detected following the manufacturer’s instructions (CSB-E08055r, SB-E04595r, CUSABIO, China). Cytokine levels in the kidney were presented by ratio of cytokine concentration and albumin concentration.

#### Histological analysis

The kidney obtained from experimental rats was cut longitudinal and fixed with 10% neutral formalin and finally embedded in paraffin. The paraffin was cut into 5 μm thick sections. Hematoxylin and eosin (H&E), periodic acid-Schiff (PAS) and Masson were used to observe the kidneys' morphological changes. Ly6G immunohistochemical staining was performed with a rabbit anti-Ly6G antibody (BS-20073R, 1:200, Bioss, China). F4/80 immunofluorescence staining was performed with rabbit anti-F4/80 antibody (70076, 1:1000, CST, USA) followed by incubation with CY3-tagged secondary antibodies (A32732, 1:400, Thermo, USA). Finally, DAPI (0100-20, SouthernBiotech, USA) was used to stain the nucleus.

For analysis, we used Pannoramic MIDI (3DHISTECH, Hungary) to scan each section under the field of light or fluorescence and circled 10 pieces of 1mm^2^ around the section randomly by CaseViewer software. Positive cells in each area were counted, and the average score or positive represented the whole section.

#### Isolation of exosomes from UC-MSCs and HEK293T

The conditioned medium of UC-MSCs and HEK293T was collected after 72 h. The medium was ultrafiltered to remove the cell debris, and then the concentrated medium was ultracentrifuged to separate the exosomes. The detailed isolation method and identification of exosomes were described in our previous work [34].

#### Cell culture

The isolation and identification of UC-MSCs refer to the previous work of our laboratory [33]. UC-MSCs were in serum-free stem cell culture medium (Lonza, MD, Walkersville) at 37 °C and 5% CO_2_ concentration. RAW264.7 and THP-1 were purchased from the China Center for Type Culture Collection (Wuhan China). RAW264.7 cells were cultured in DMEM medium containing 10% fetal bovine serum (FBS; Hyclone, USA), THP1 were cultured in RPMI 1640 medium with 10% FBS at 37 °C and 5% CO_2_ concentration in a humidified atmosphere. For co-culture experiment, macrophages were seeded into a six-well plate. The next day, the UC-MSC-CM was placed into the six-well plate with the macrophages that were initially seeded. Co-cultures were incubated for 24 h with 100 ng/ml LPS & 30 ng/ml IFN-γ [35].

#### RNA extraction and real-time PCR analysis

Total RNA (> 200 nt) was extracted from renal tissue or cultured cells with Trizol (15596-018, Invitrogen, USA), and small RNA (20–200 nt) were extracted by RNAiso for Small RNA (9353A, Takara, China) according to the procedure recommended by the manufacturer. RNA was reversed transcribed into cDNA with a reagent kit (RR047B and 638315, Takara, China). Real-time PCR was performed using SYBR Green reagent (CW0957W, CWBIO, China) on the Real-time PCR System (CFX connect, Bio-Rad, USA). The relative gene and miRNAs expression were determined after normalization to the endogenous housekeeping gene GAPDH and U6 with 2^−ΔΔCt^ method. Primer sequences are shown in Additional file 1: Table S1.

#### RNA high-throughput sequencing analysis

Tissue RNA was extracted from control and DN kidney, and exosome RNA was extracted from HEK293T- and UC-MSCs-derived exosomes. Samples were submitted in biological triplicate to the MajorBio Platform for quality control, library creation, and high-throughput sequencing.

After total RNA extraction, purity, concentration, and integrity of RNA were determined by Nanodrop (Thermo Fisher Scientific, USA), and Agilent 2100 Bioanalyzer (Agilent Technologies, USA), respectively. For miRNA sequencing, sequencing libraries were generated using NEB Next Ultra small RNA Sample Library Prep Kit for Illumina (NEB, USA) according to manufacturer’s instructions. Finally, PCR products were purified and library quality was assessed on the Agilent Bioanalyzer 2100 system (Agilent Technologies, USA). After quality assessment, sequencing of all libraries was performed by the Illumina Hiseq 2500 platform (Illumina, USA). The raw reads were trimmed and cleaned by removing sequences shorter than 18 nt or longer than 30 nt. Simultaneously, Q20, Q30, GC-content, and sequence duplication level of clean data was calculated. All the downstream analyses were based on clean data with high quality. Differential miRNA expression analyses were performed using the DESeq R package (v1.18.0, EMBL Heidelberg, Germany).

#### Luciferase reporter assay

The predicted 3′-UTR sequence of TRAF6 interacting with miR-146a-5p and mutated sequences within the predicted target sites were synthesized and inserted into the pmirGLO control vector (E1330, Promega, USA). HEK293T were transfected with 7.5 pmol miR-146a-5p or negative control and 2.5 μg of the wild-type or mutant 3′-UTR plasmid by Lipofectamine 2000 (11668027, Invitrogen, USA). After 48 h of transfection, luciferase activity of cells was measured using a Dual Luciferase Assay Kit (FR201-01, TransGen, China). Renilla luciferase was used to normalize the value of firefly luciferase.

#### microRNA and siRNA transfection

Both of the microRNA and small interfering RNA (siRNA) were synthesized by the RIBBIO company in Guangzhou, China. The mimic, inhibitor and their NC of miR-146a-5p were transfected into UC-MSCs at a concentration of 100 nM using lipofectamine RNAiMAX (13778150, Invitrogen, USA) as instructed by the manufacturer’s protocols. Both RAW264.7 and THP1 were transfected with siRNA at a concentration of 100 nM using Lipofectamine 2000 (11668019, Invitrogen, USA) as instructed by the manufacturer’s protocols. miR-146a-5p mimic, miR-146a-5p inhibitor, and TRAF6 siRNA sequences are shown in Additional file 1: Table S2 and Table S3.

### Western blot analysis

Cells and renal tissues were lysed in ice-cold RIPA Lysis buffer (P0013C, Beyotime, China) supplemented with protease inhibitor cocktail and protein concentration was determined by BCA assay. Proteins were separated by sodium dodecyl sulfate–polyacrylamide gel electrophoresis (SDS-PAGE) and then transferred into a PVDF membrane. Antibodies were used in western blot as follows: anti-β-actin (60004-1-Ig, 1:5000, Proteintech, China), anti-Arg1 (16001-1-AP, 1:5000, Proteintech, China), anti-iNOS (18985-1-AP, 1:2000, Proteintech, China), anti-TRAF6(abs145675, 1:1000, Absin, China), anti-pSTAT1(abs130924, 1:1000, Absin, China).

#### Statistical analysis

Data are expressed as means ± SD. The two-tailed Student’s t-test was employed for comparisons between two groups, and one-way ANOVA were performed for comparisons of data with more than two groups. *P* < 0.05 was considered statistically significant. In vitro experiments were assessed in at least three independent experiments. All analyses were carried out by GraphPad Prism 5.0.

## Results

### UC-MSC administration ameliorates renal injury in DN rats

**Fig. 1:**
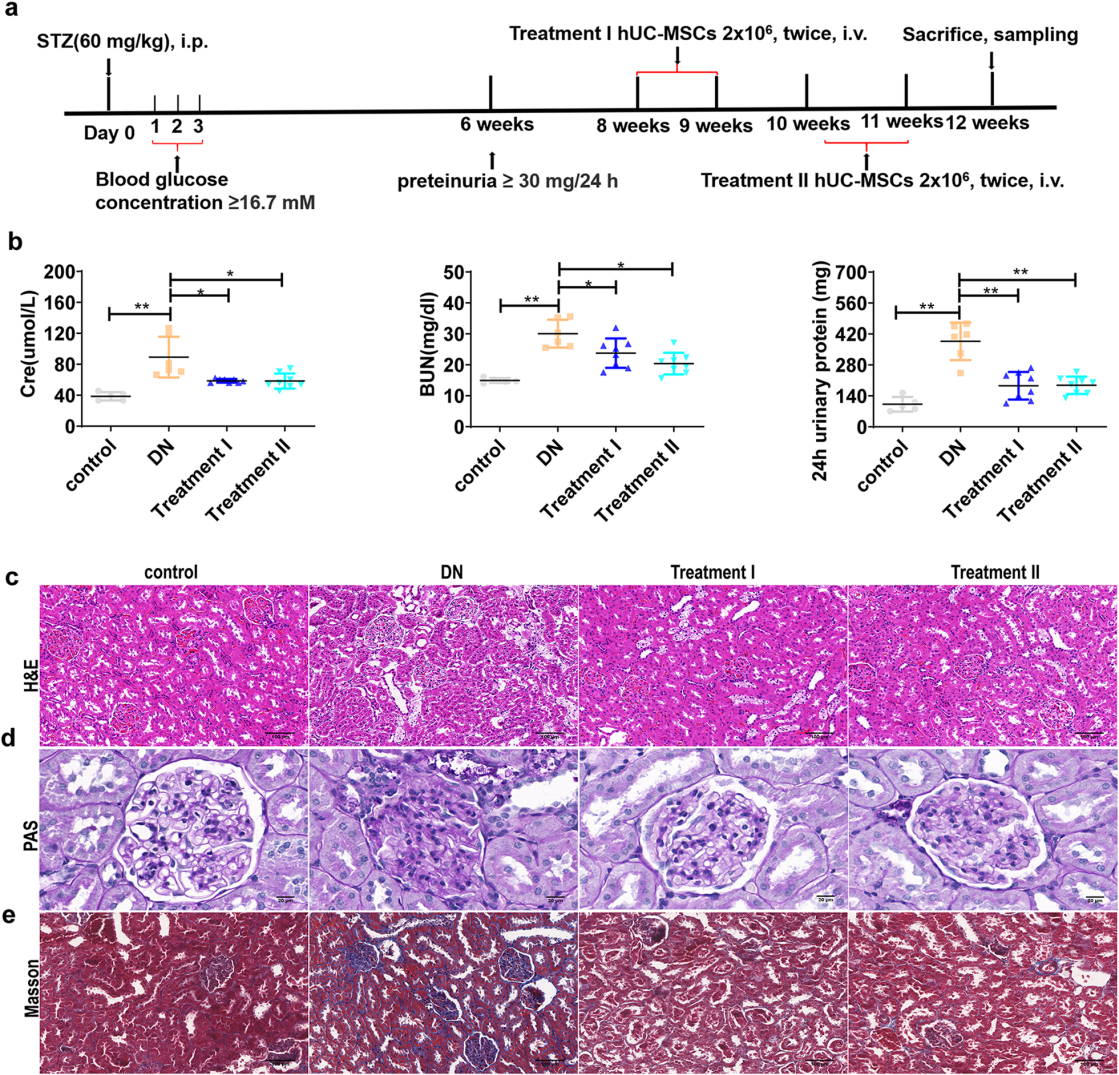
UC-MSC administration ameliorates renal injury in DN rats. Rats were exposed to 60 mg/kg STZ by intraperitoneal to induce DN model. UC-MSCs (2 × 10^6^/500 μl) were administrated via the tail veil to assess the therapeutic effect. **a** Flowchart and timetable of rat treatment from day 0 to week 12. **b** Renal function in DN rats with or without UC-MSC administration was determined by measuring serum creatinine, serum urea nitrogen, 24 h total urine protein and creatinine clearance rate at week 12, *n* = 5–8 rats/group. H&E staining (**c**), PAS staining (**d**) and Masson staining (**e**) in the kidneys to observe histological changes, *n* = 3 rats/group, Scale bar: 100 µm in H&E and Masson, Scale bar: 20 µm in PAS. Data presented as mean ± SD of individuals included in each group. *P < 0.05, **P < 0.01

To explore the therapeutic effect of UC-MSCs, we established a STZ-induced DN model of rats. The experimental design was shown in Fig. 1a, and the difference between the two treatment groups was that UC-MSCs were transplanted at different time points of DN. We analyzed renal function and pathological changes among control rats, DN rats, and UC-MSC-treated DN rats. All of the experimental rats were induced to become diabetes and developed into DN after intraperitoneal injection of STZ. Compared with DN rats, UC-MSC-treated DN rats showed a significant decrease in serum creatinine (CRE), serum urea nitrogen (BUN) and 24 h total urinary protein (Fig. 1b). Moreover, UC-MSC administration attenuated tubular dilatation, massive accumulation of inflammatory cells in the interstitial area, renal glomerular hypertrophy and sclerosis, and renal interstitial fibrosis in the DN kidney (Fig. 1c–e). These results indicate that UC-MSCs ameliorate renal injury in DN rats, and transplantation of UC-MSCs at different time points of DN has a consistent therapeutic effect.

### UC-MSC administration inhibits renal inflammation in DN rats

**Fig. 2:**
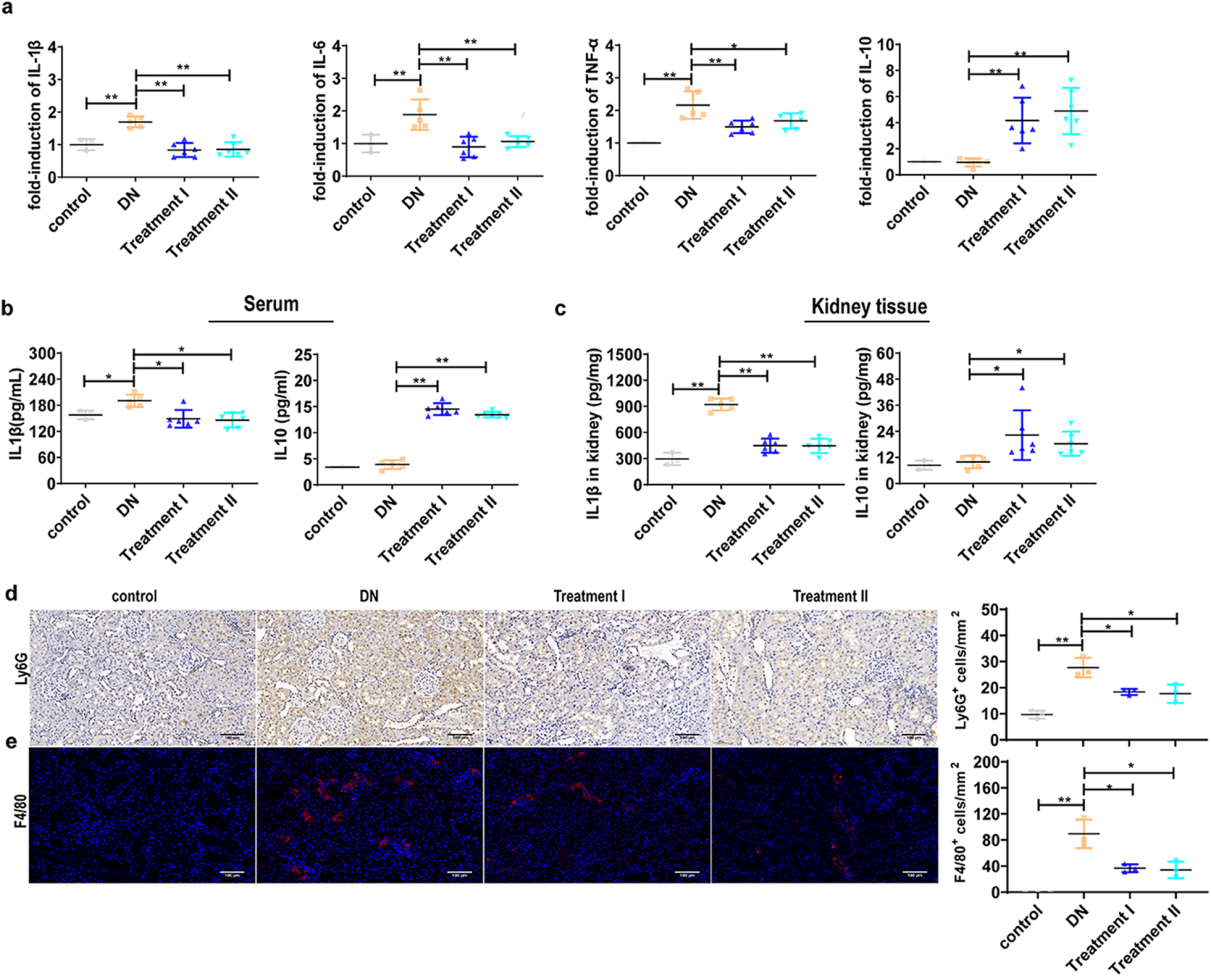
UC-MSC administration inhibits renal inflammation in DN rats. **a** Relative mRNA expression of IL-6, IL-1β, TNF-α, and IL-10 in the kidney from DN rats with or without UC-MSC administration. **b** Concentration of IL-1β in the kidney and serum as measured by ELISA. **c** Concentration of IL-10 in the kidney and serum as measured by ELISA (*n* = 5–8 rats/group). **d** Infiltrating neutrophils in the kidney as represented by Ly6G immunohistochemistry staining. **e** Infiltrating macrophages in the kidney as represented by F4/80 immunofluorescence staining. *n* = 3 rats/group, Scale bar: 100 µm. Data presented as mean ± SD of individuals included in each group. *P < 0.05, **P < 0.01

To investigate whether UC-MSCs modulate the inflammatory response in the DN rats, we determined the levels of pro- and anti-inflammatory cytokines and quantified inflammatory cell infiltration in the DN rats with or without UC-MSC treatment. Unlike in DN group which showed elevated mRNA levels of pro-inflammatory IL-1β, IL-6 and TNF-α, UC-MSC treatment in rats significantly reduced the expression of pro-inflammatory cytokines but augmented anti-inflammatory IL-10 expression (Fig. 2a). Consistent with this data, decreased IL-1β production and increased IL-10 production (Fig. 2b, c) were observed in kidneys and serum from UC-MSC-treated DN rats, as compared with untreated controls. Moreover, UC-MSC administration resulted in a significant decreased accumulation of Ly6G^+^ neutrophils (Fig. 2d) and F4/80^+^ macrophages (Fig. 2e), as determined by immunohistochemistry and immunofluorescence staining. These results indicate that UC-MSC administration reduces systemic and local renal inflammation in DN rats.

### UC-MSCs shift macrophage polarization toward an M2 phenotype in vivo and in *vitro*

**Fig. 3:**
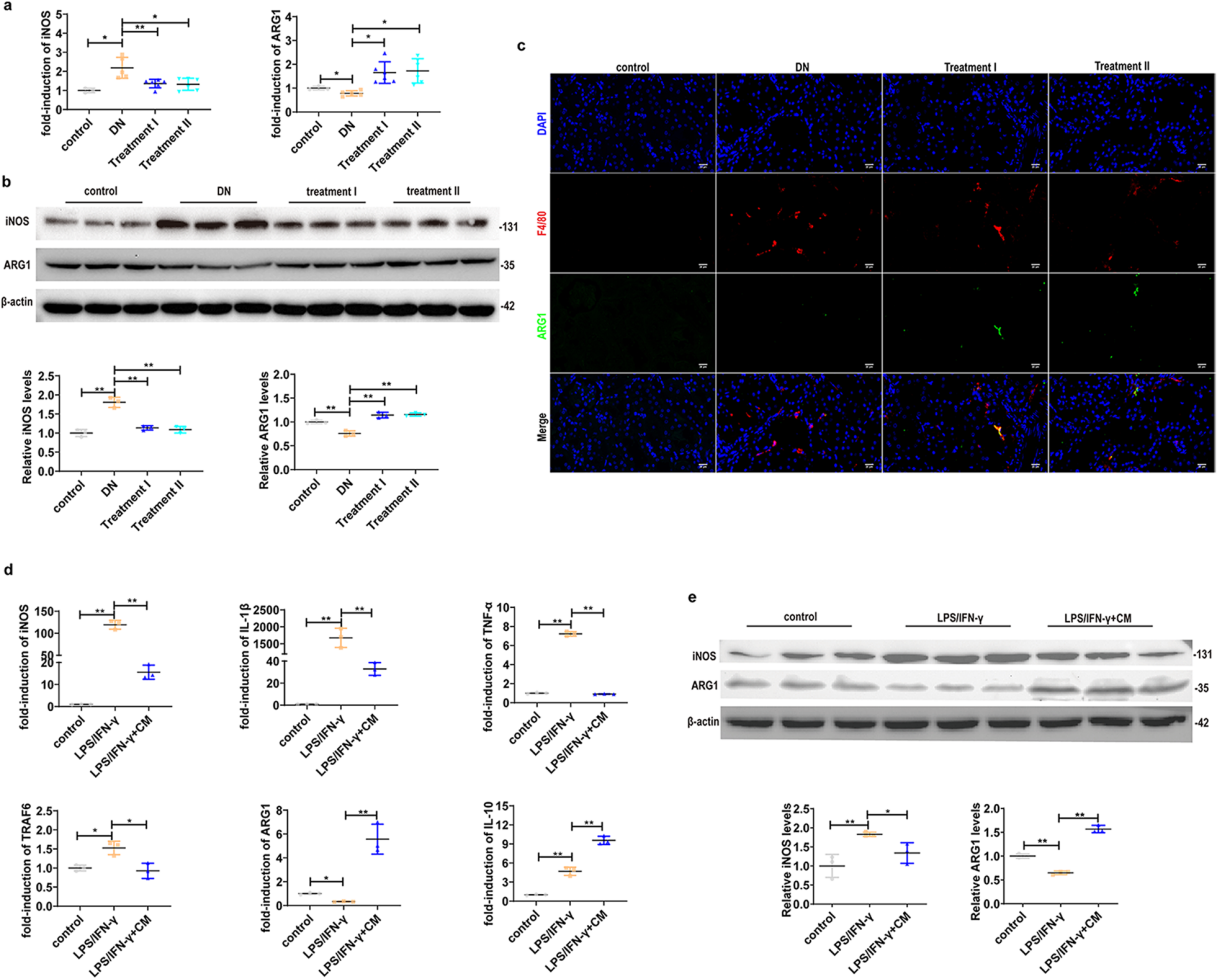
UC-MSCs shift macrophage polarization toward an M2 phenotype. **a** Relative mRNA expression of iNOS and ARG1 in the kidney from DN rats with or without UC-MSC administration, *n* = 5–8 rats/group. **b** Relative protein expression of iNOS and ARG1 in the kidney, *n* = 3 rats/group. **c** Representative immunofluorescence staining for the macrophage marker F4/80 (red) and ARG1 (green) colocalization in the kidney from DN rats with or without UC-MSC administration, Scale bar: 20 µm. **d** RAW264.7 cell were stimulated with LPS/IFN-γ and treated with UC-MSC- conditioned medium (UC-MSC-CM) to explore the effect of UC-MSC-CM on regulation of macrophage polarization in vitro*.* Relative mRNA expression of inflammatory cytokines and macrophage markers including iNOS, IL-1β, TNF-α, TRAF6, ARG1, and IL-10. **e** Relative protein expression and semi-quantitative analysis of M1/M2 macrophage markers (iNOS and ARG1) in RAW264.7. Data presented as mean ± SD of individuals included in each group. Results in vitro are representative of three independent experiments. *P < 0.05, **P < 0.01

Since macrophage M1 activation is implicated in the pathogenesis of chronic inflammatory diseases including DN [36], we next determined whether UC-MSCs may regulate macrophage polarization in DN rats. The results showed that administration of UC-MSCs increased arginase1 (ARG1) expression, an M2 macrophage marker, but decreased inducible nitric oxide synthase (iNOS) expression, an M1 macrophage marker, at the mRNA and protein levels (Fig. 3a, b). Moreover, using double immunofluorescence staining, we found that UC-MSC administration enhanced M2 macrophage ARG1 expression (Fig. 3c).

Next, we used UC-MSC-conditioned medium (UC-MSC-CM) to treat the macrophage cell line RAW264.7 in response to stimulation with LPS (100 ng/ml) and IFN-γ (30 ng/ml). Clearly, UC-MSC-CM treatment resulted in significant decrease in the expressions of iNOS, IL-1β, TNF-α, and TRAF6, but increase in ARG1 and IL-10 expression in LPS/IFN-γ-stimulated RAW264.7, as compared with untreated controls (Fig. 3d, e). To further demonstrate the effect of UC-MSCs on macrophage polarization, we used human macrophage cell line THP1. Similarly, UC-MSC-CM treatment markedly reduced iNOS, IL-1β, TNF-α, TRAF6 and p-STAT1 expression, but increased ARG1 and IL-10 expression in LPS/IFN-γ-stimulated THP1 (Additional file 1: Fig. S1). Taken together, these results suggest UC-MSCs regulate macrophage polarization and function during inflammatory response via a paracrine mechanism.

### High-throughput sequencing analysis of miRNA profile in the DN kidney and UC-MSCs

**Fig. 4:**
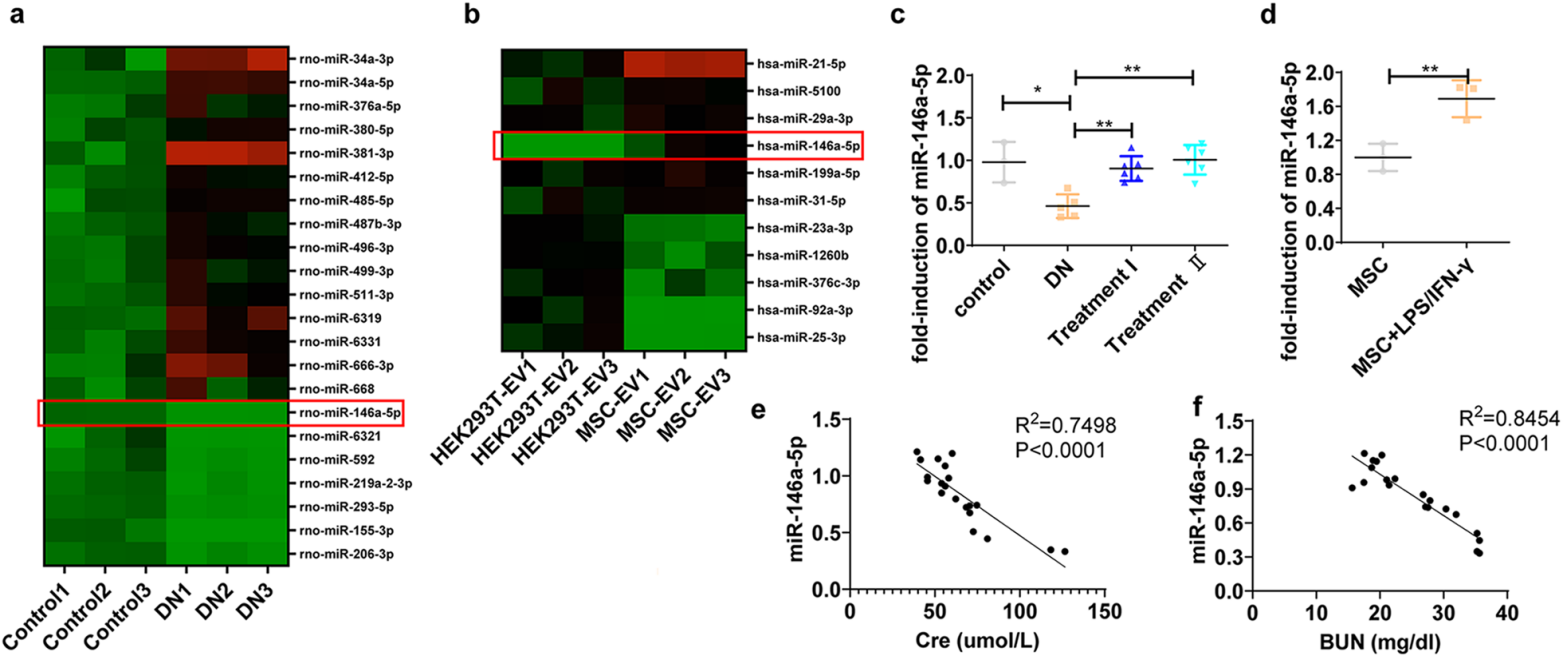
High-throughput sequencing analysis of miRNA profile in the DN kidney and UC-MSCs. miRNAs high-throughput sequencing was performed to identify specific miRNAs which were abnormal expression in the DN kidney and abundant in UC-MSCs derived exosomes. **a** The heatmap of down- or up-regulated miRNAs identified by RNA high-throughput sequencing in the control and DN kidney, *n* = 3 rats/group. **b** The heatmap of down- or up-regulated miRNAs identified by high-throughput sequencing in the HEK293T- and UC-MSC-derived exosomes, *n* = 3 samples/group. **c** The expression of miR-146a-5p in the kidney from DN rats with or without UC-MSC administration, *n* = 5–8 rats/group. **d** The expression of miR-146a-5p in UC-MSCs with or without LPS(100 ng/ml)/IFN-γ(30 ng/ml) stimulation. **e** Correlation analysis of miR-146a-5p and serum Cre in rats. **f** Correlation analysis of miR-146a-5p and serum BUN in rats, *n* = 20 rats. Data presented as mean ± SD of individuals included in each group. Results in vitro are representative of three independent experiments. *P < 0.05, **P < 0.01

To explore the communication between miRNAs and macrophage polarization in DN, we examined the miRNAs in kidney tissues of normal and DN rats using high-throughput sequencing. Moreover, we measured the expression levels of various miRNAs between exosomes derived from HEK293T and UC-MSCs. Analysis of differentially expressed miRNAs revealed that miR-146a-5p was significantly down-regulated in the DN kidney and abundant in UC-MSC-derived exosomes (Fig. 4a, b). Further validation of miR-146a-5p expression in the kidney by qRT-PCR confirmed the expression of miR-146a-5p was down-regulated in the DN rats and restored in the UC-MSC-treated DN rats (Fig. 4c). We also found that miR-146a-5p expression was significantly increased in LPS/IFN-γ-stimulated UC-MSCs, as compared with unstimulated controls. (Fig. 4d). More importantly, the expression of miR-146a-5p in the kidney was negatively correlated with the concentration of Cre and BUN in serum (Fig. 4e, f). These results reveal the potential function and candidate biomarker attributes of miR-146a-5p in DN, and suggest that UC-MSC-derived miR-146a-5p may play an important role in protection against DN.

### UC-MSCs-derived miR-146a-5p targets TRAF6 and facilitates M2 macrophage polarization

**Fig. 5:**
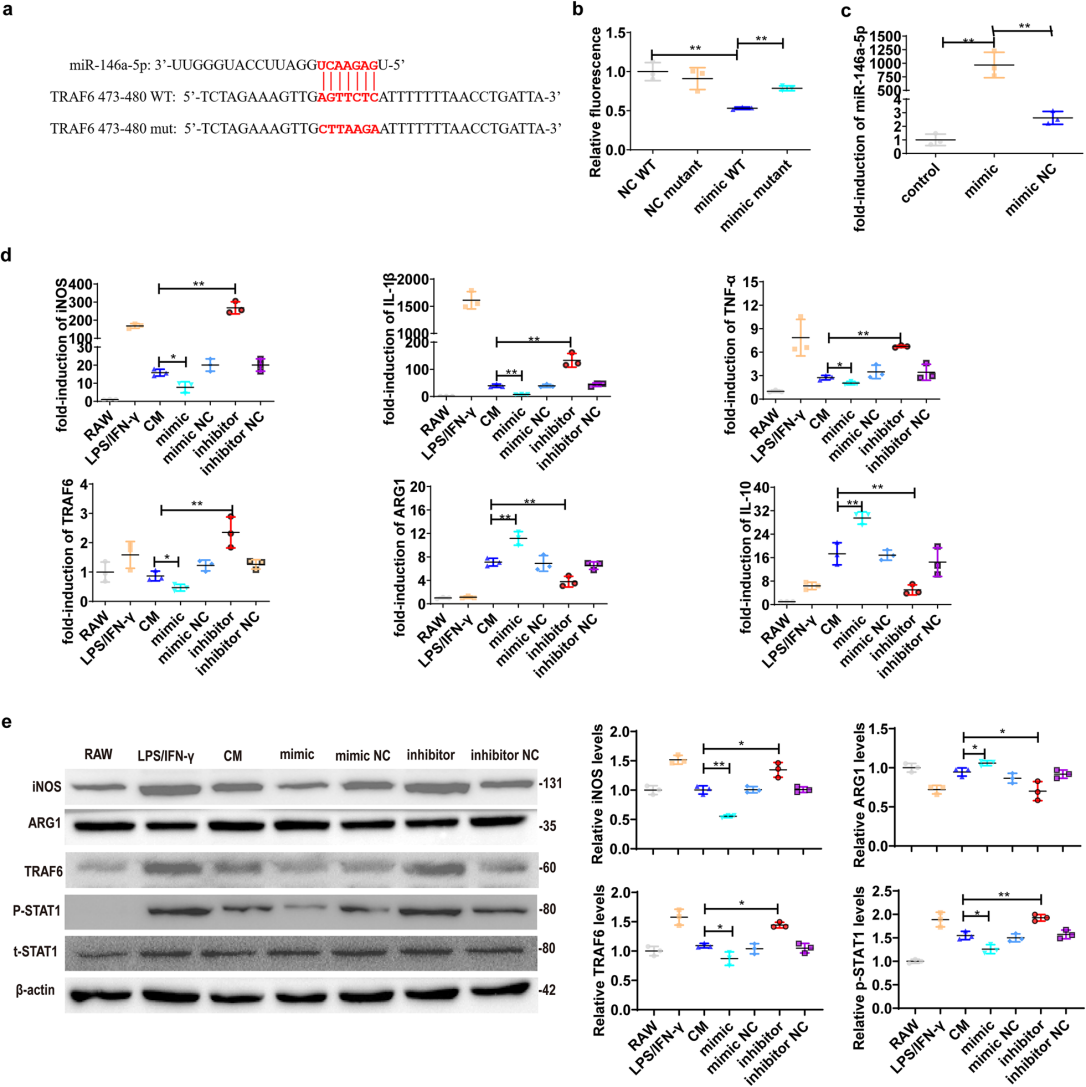
UC-MSC-derived miR-146a-5p targets TRAF6 and facilitates M2 macrophage polarization in RAW264.7. **a** TargetScan predicted miR-146a-5p binding sites in the 3′UTR at 473–480 sites of TRAF6 and the corresponding designed mutation site. **b** Luciferase activity was measured in HEK293T cells were co-transfected with miR-146a-5p mimic or miR-146a-5p NC and luciferase reporter plasmids containing wild-type or mutant. **c** The expression of miR-146a-5p in control UC-MSCs, miR-146a-5p mimic transfected UC-MSCs and miR-146a-5p mimic NC transfected UC-MSCs. The miR-146a-5p mimic, miR-146a-5p inhibitor and their negative controls were transfected into UC-MSCs, and the CM was collected to treat RAW264.7. **d** Relative mRNA expression of inflammatory cytokines and M1/M2 macrophage markers including iNOS, IL-1β, TNF-α, TRAF6, ARG1, and IL-10 in the control RAW264.7, LPS/IFN-γ-stimulated RAW264.7 and LPS/IFN-γ-stimulated RAW264.7 treated with the indicated CM. **e** Relative protein expression and semi-quantitative analysis of iNOS, ARG1, TRAF6, and p-STAT1 in RAW264.7. Data presented as mean ± SD in each group. Results in vitro are representative of three independent experiments. *P < 0.05, **P < 0.01

To identify the mechanism how miR-146a-5p regulates macrophage polarization during DN, we performed bioinformatics analyses according to TargetScan and microRNA.org. The web-based prediction software for targets of miRNAs, TRAF6 3′-UTR region contains the putative binding sequence of miR-146a-5p, could be a potential target gene of miR-146a-5p (Fig. 5a). Using the dual-luciferase assay, we observed that miR-146a-5p mimics significantly suppressed luciferase activity when wide type (WT) 3′UTR of TRAF6 was inserted downstream of the luciferase report plasmid, as compared with mutant 3′UTR (Fig. 5b).

Moreover, we transfected miR-146a-5p mimic, miR-146a-5p inhibitor, and their negative control (NC) into UC-MSCs, and collected UC-MSC-CM to treat LPS/IFN-γ-stimulated RAW264.7. As expected, transfection with miR-146a-5p mimic obviously increased miR-146a-5p expression in UC-MSCs, as compared with control UC-MSCs (Fig. 5c). We also observed that CM derived from miR-146a-5p mimic-transfected UC-MSCs markedly decreased the expressions of iNOS, IL-1β, TNF-α, TRAF6 and p-STAT1, but significantly increased ARG1 and IL-10 expressions compared to control UC-MSCs-CM. However, CM derived from miR-146a-5p inhibitor-transfected UC-MSCs exerted the opposite results (Fig. 5 d, e). Similar results were observed in human macrophage cell line THP1. Transfection with miR-146a-5p mimic leaded to promote M2 macrophage polarization, and transfection with miR-146a-5p inhibitor resulted in facilitating M1 macrophage polarization in THP1 cells (Additional file 1: Fig. S1). Together, these results demonstrate that miR-146a-5p contributes to UC-MSCs-mediated M2 macrophage polarization.

### TRAF6 is required for UC-MSC-miR-146a-5p-mediated M2 macrophage polarization

**Fig. 6:**
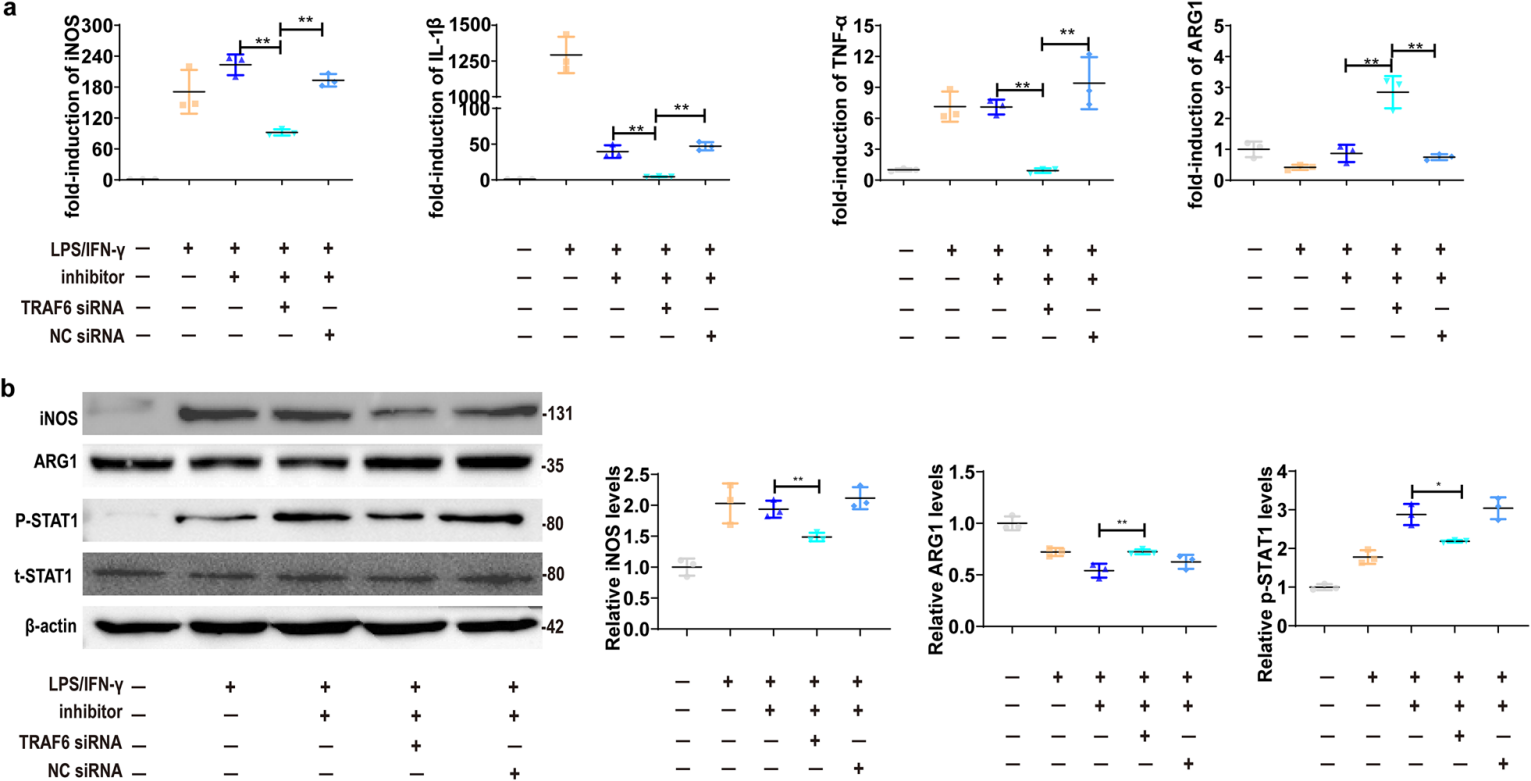
TRAF6 is required for UC-MSCs-derived miR-146a-5p-mediated M2 macrophage polarization. RAW264.7 cells were transfected with TRAF6 siRNA or negative control (NC) siRNA, and then treated with CM derived from miR-146a-5p inhibitor transfected-UC-MSCs. **a** Relative mRNA expression of inflammatory cytokines and M1/M2 macrophage markers including iNOS, IL-1β, TNF-α, and ARG1 in RAW264.7. **b** Relative protein expression and semi-quantitative analysis of INOS, ARG1, and p-STAT1 in RAW264.7. Data presented as mean ± SD in each group. Results in vitro are representative of three independent experiments. *P < 0.05, **P < 0.01

To further elucidate whether miR-146a-5p regulated macrophage polarization by targeting TRAF6, we used TRAF6 siRNA to knockdown TRAF6 in macrophage cell line and cocultured with miR-146a-5p inhibitor transfected-UC-MSCs. The knockdown efficiency of TRAF6 in RAW264.7 and THP1 was detected by qRT-PCR (Additional file 1: Fig. S2a, c) and western blot analyze (Additional file 1: Fig. S2b, c). We found that TRAF6 downregulation resulted in decreased expressions of iNOS, IL-1β, TNF-α and p-STAT1, and elevated ARG1 expression in RAW264.7 cells treated with CM derived from miR-146a-5p inhibitor-transfected UC-MSCs (Fig. 6a, b). Similar results were observed in THP1, indicating the elevated anti-inflammatory factors and reduced pro-inflammatory factors in the presence of miR-146a-5p inhibitor when TRAF6 was silenced (Additional file 1: Fig. S2e, f). These results demonstrate that TRAF6 plays essential role in miR-146a-5p-regulated macrophage polarization.

### MiR-146a-5p-modified UC-MSCs enhance the efficacy of anti-inflammation and renal function improvement in DN rats

**Fig. 7:**
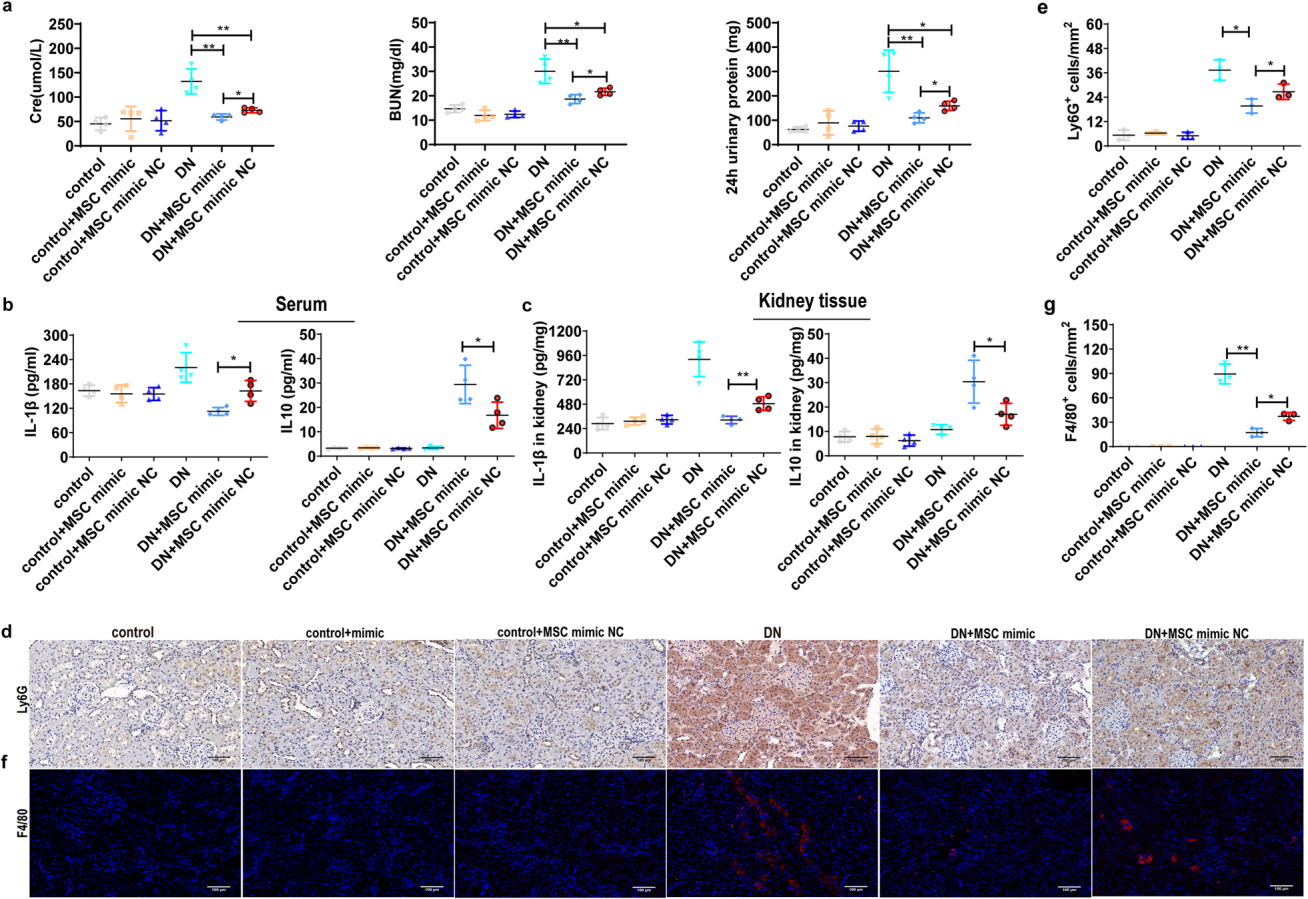
miR-146a-5p-modified UC-MSCs enhance the efficacy of anti-inflammation and renal function improvement in DN rats. UC-MSCs and miR-146a-5p-modified UC-MSCs were intravenously administrated to explore the renal protection and macrophage polarization regulation of miR-146a-5p on DN rats. **a** Renal function was assessed by measuring serum creatinine, serum urea nitrogen, 24 h total urine protein and creatinine clearance rate in rats. **b**, **c** Concentration of IL-1β and IL-10 in the kidney and serum from each group of rats as measured by ELISA, *n* = 4 rats/group. **d**, **e** Infiltrated neutrophils in the kidney as represented by Ly6G immunohistochemistry staining. **f**, **g** Infiltrated macrophages in the kidney as represented by F4/80 immunofluorescence staining, *n* = 3 rats/group, Scale bar: 100 µm

**Fig. 8:**
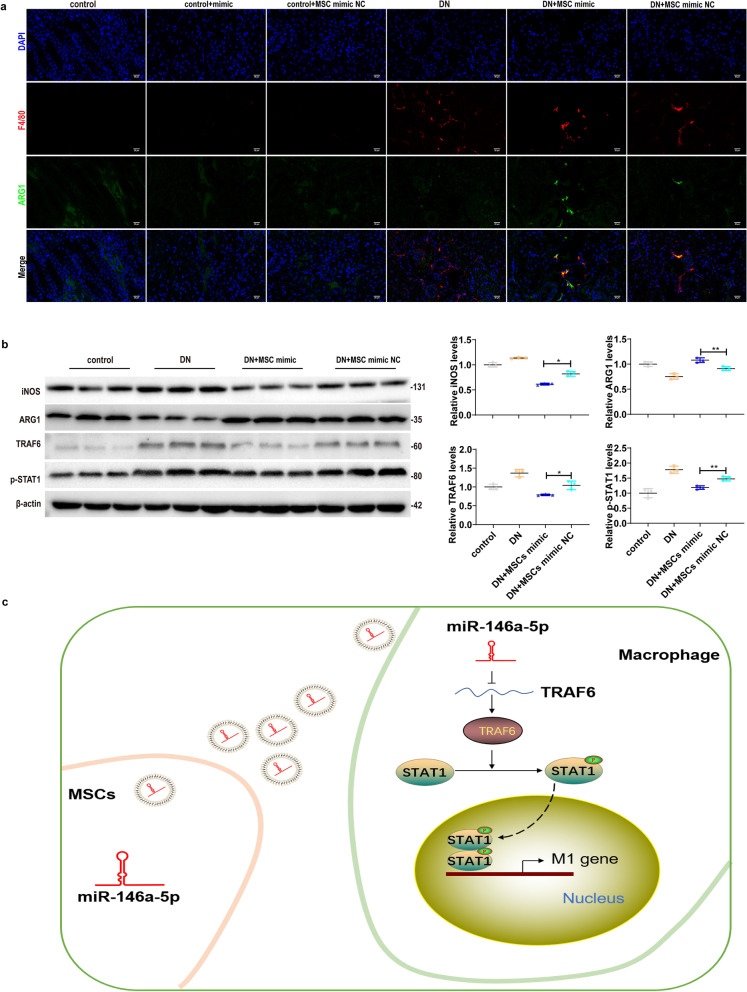
miR-146a-5p-modified UC-MSCs facilitate M2 macrophage polarization in DN rats. **a** Representative immunofluorescence staining for the macrophage marker F4/80 (red) and ARG1 (green) colocalization in the kidney, Scale bar: 20 µm. **b** Relative protein expression and semi-quantitative analysis of iNOS, ARG1, TRAF6, and p-STAT1 in the kidney. Data presented as mean ± SD. **c** UC-MSCs, overexpressing miR-146a-5p, can be delivered to damaged kidneys to inhibit M1 macrophage activation but facilitate M2 macrophage polarization by targeting TRAF6-STAT1 signaling pathway, leading to reduced inflammation and renal injury in DN rats. Data presented as mean ± SD of individuals included in each group. *P < 0.05, **P < 0.01

To further confirm the regulatory role of miR-146a-5p in macrophage polarization in vivo, we transfected miR-146a-5p mimic or miR-146a-5p mimic NC into UC-MSCs, and transplanted the modified-UC-MSCs into DN rats via the tail vein*.* Subsequently, we determined the levels of inflammatory cytokines, quantified inflammatory cell infiltration and M1/M2 macrophage marker expression in the experimental rats. The results showed that administration of miR-146a-5p mimic-transfected UC-MSCs in DN rats obviously reduced Cre, BUN and 24 h total urinary protein compared to miR-146a-5p mimic NC-transfected UC-MSCs (Fig. 7a). Moreover, miR-146a-5p mimic-transfected UC-MSC administration further attenuated kidney pathological damage (Additional file 1: Fig. S3). As compared with miR-146a-5p mimic NC-transfected UC-MSC administration in DN rats, decreased IL-1βand increased IL-10 production (Fig. 7b, c) were observed in kidney tissues and serum from miR-146a-5p mimic-transfected UC-MSCs-treated DN rats. miR-146a-5p mimic-transfected UC-MSC administration in DN rats also resulted in a significant decreased accumulation of Ly6G^+^ neutrophils (Fig. 7d, e) and F4/80^+^ macrophages (Fig. 7f, g), as determined by immunohistochemistry and immunofluorescence staining. Moreover, using double immunofluorescence staining, we found that miR-146a-5p mimic-transfected UC-MSC administration enhanced M2 macrophage ARG1 expression in DN rats (Fig. 8a). Notably, western blot analysis indicated the expression of iNOS, TRAF6 and p-STAT1 were significantly decreased, but ARG1 expression was increased in miR-146a-5p mimic-transfected UC-MSC-treated DN rats (Fig. 8b). Collectively, these results reveal that miR-146a-5p-modified UC-MSCs enhance the efficacy of renal function improvement and regulation of macrophage polarization in DN.

## Discussion

DN is the most prevalent chronic renal disease and the major cause of ESRD. Inflammation is considered the key mediators of the development and progression of renal injury in DN [9, 10]. In this study, we demonstrated that UC-MSC administration significantly improved renal function of DN rats. Importantly, UC-MSCs facilitated M2 macrophage differentiation, accompanied by reduced systemic and local renal inflammation in DN rats. We further identified that miR-146a-5p was dramatically decreased and negatively correlated with renal injury in DN rats. Furthermore, UC-MSC-derived miR-146a-5p shifted M2 macrophage polarization by targeting TRAF6-STAT1 signaling pathway. Finally, we proved miR-146a-5p-modified UC-MSCs enhanced the efficacy of anti-inflammation and renal function improvement in DN rats.

Data from other groups as well as our previous studies revealed that MSCs represent promising cell-based therapeutics for tissue injury and disease, including chronic kidney disease [33, 38, 39]. However, the exact molecular mechanisms for MSC-based therapy for DN have not been fully elucidated. It is well documented that MSCs may provide a means for recapitulating multiple mechanisms to repair tissue injury, including immunomodulation, antioxidant, autophagy, and anti-fibrosis [40–42]. Notably, the potent immunomodulatory and anti-inflammatory properties of MSCs are currently the focus of intensive studies in graft enhancement, tissue protection, and regenerative medicine [43]. Multiple lines of evidence are presented to show that inflammation is a key pathogenic factor during the pathogenesis of DN, and that imbalance of M1/M2 macrophages plays a central role in inflammation [13]. Here we discovered that UC-MSCs reduced systemic and local renal inflammation, at least in part, through facilitating macrophage polarization from a pro-inflammatory M1 to an anti-inflammatory M2 phenotype. It should be noted that although there are studies exploring the clinical effects of MSCs on DN, the timing and therapeutic window for cell therapy remains elusive. In this study, DN rats were treated with two injections of UC-MSCs at week 7 and 8, or week 9 and 10, respectively. Our results showed that administration of UC-MSCs at these two different time points of DN has similar protection against renal injury and inflammation, demonstrating the therapeutic efficacy of UC-MSC injection in different time points of DN.

miRNAs carried by circulating exosomes have been widely proved as significant contributors to tissue homeostasis and feasible therapeutics for various diseases [44, 45]. Indeed, miRNAs have recently emerged as important regulators and been implicated as the key regulators of different molecules involved in the DN network [37]. However, the role of miRNAs in the regulation of inflammation during DN is not fully understood. Importantly, MSCs have been demonstrated to be a safe and effective delivery vehicle for therapeutic miRNA treatment, due to their ability to specifically target immune disorder, inflammation and fibrosis [46, 47]. In this study, through high-throughput RNA sequencing, we found that UC-MSC-exosomes had a specific miRNA abundance signature that was different from that of HEK293T-exosomes. In particular, we identified that UC-MSC-derived miR-146a-5p played a critical role in macrophage polarization during DN. Previous studies reported that miR-146a-5p was shown to be involved in the regulation of inflammatory response [48–50]. Here, our findings demonstrate that UC-MSCs exert a protective effect on DN through delivery of miR-146a-5p, resulting in suppression of inflammation. These studies suggest that miR-146a-5p might act as an important anti-inflammatory noncoding RNA modulator of DN and other inflammatory diseases. More importantly, our study revealed a novel role of exosomal miRNA in UC-MSC-mediated therapy in DN. However, further work is needed to evaluate the therapeutic efficacy of UC-MSC-derived exosomes in DN rats.

TRAF6 contains ring finger domains that are commonly found in ubiquitin ligases (E3), which can be conjugated to another molecule to form different polyubiquitin chain [51]. It delivers the signaling through ubiquitination and interaction with transforming growth factor-β-activated kinase 1 [52], which activates the nuclear factor-κB (NF-κB) signaling pathway [53, 54]. In the context of the immune system, TRAF6-mediated signals have proven critical for the development, homeostasis, and activation of innate immune cells, including macrophages [55]. TRAF6 had also been reported as a potential therapeutic target to normalize inflammation in DN, while knocking down the expression of TRAF6 in kidneys of diabetic mice would attenuate renal inflammation [56]. In addition, STAT proteins are identified as a family of latent cytoplasmic transcription factors and play a critical role in transducing signals from various cytokines to achieve distinct transcriptional outcomes [57]. Recently, emerging evidence suggests that STATs play important roles in macrophage polarization [58]. Among the family members, evidence suggested that IFN-γ activates JAK-STAT1 signaling and promotes STAT1 phosphorylation, which leads to the M1 macrophage polarization [59]. Luu and colleagues [60] also demonstrated cross-talk between the TLR and JAK/STAT signaling pathways with direct recruitment of STAT1 by TRAF6. In keeping these studies, our data suggested that TRAF6-STAT1 signaling was involved in renal inflammation in DN rats. More importantly, we identified that UC-MSCs-derived miR-146a-5p targeted the TRAF6-STAT1 pathway to suppress kidney inflammation and restore renal function through facilitating M2 macrophage polarization.

With the ability of immunomodulation and anti-inflammation, MSC-based therapy has been applied in various immune- and inflammation-mediated diseases [61]. However, a number of phase III clinical trials of MSC immunotherapy were unable to meet the primary endpoints because of the low immunoregulatory efficacy of engrafted cells [62]. In the quest to circumvent these challenges, several modification techniques have been applied to improve the therapeutic efficacy of MSCs. For instance, hepatocyte growth factor or vascular endothelial growth factor overexpressing MSCs maximized MSC-based myocardial salvage after acute myocardial infarction [63]. CXCR4 receptor overexpression in MSCs improved MSC homing and facilitated treatment of acute lung injury in rats [64]. In this study, we demonstrated that miR-146a-5p-modified UC-MSCs promoted M2 macrophage polarization and enhanced protection against renal injury in DN rats, which further proved the key role of miR-146a-5p in macrophage polarization during DN.

## Conclusion

we identify the role of miR146a-5p/TRAF6 signaling in controlling macrophage polarization in UC-MSC-mediated immune regulation. Our findings demonstrate a potential beneficial effect of UC-MSC administration on the pathophysiology of STZ-induced DN. Importantly, the present study suggests that miR146a-5p-modifed UC-MSCs enhance protection against renal injury in DN through facilitating M2 macrophage polarization by targeting TRAF6-STAT1 signaling, which may offer new therapeutic approaches for DN currently lacking effective treatment. Further studies are required to optimize dose, timing, and duration of UC-MSCs and to delineate the multiple molecular mechanisms underlying UC-MSC protection against DN.

## Supplementary Information

**Additional file 1: Fig. S1.** UC-MSC-derived miR-146a-5p targets TRAF6 and facilitates M2 macrophage polarization in THP1. **Fig. S2.** TRAF6 is required for UC-MSCs-derived miR-146a-5p-mediated M2 macrophage polarization in THP1. **Fig. S3.** miR-146a-5p modification in UC-MSCs enhanced the efficacy renal pathological improvement. **Table S1.** Primers for qRT-PCR. **Table S2.** The sequence of miRNAs. **Table S3.** The target sequence of siRNAs

## Data Availability

All data generated and/or analyzed during this study are included in this published article.

## Abbreviations

DN: Diabetic nephropathy
ESRD: End-stage renal disease
MSCs: Mesenchymal stem cells
UC-MSCs: Human umbilical cord-derived MSCs
miRNAs: MicroRNAs
STZ: Streptozotocin
H&E: Hematoxylin and eosin
PAS: Periodic Acid-Schiff
CRE: Creatinine
BUN: Urea nitrogen
IL-6: Interleukin 6
IL-1β: Interleukin 1β
TNF-α: Tumor necrosis factor-α
IL-10: Interleukin 10
ARG1: Arginase1
iNOS: Inducible nitric oxide synthase
UC-MSC-CM: UC-MSC-conditioned medium
NC: Negative control
RAW264.7: Mouse mononuclear macrophage leukemia cells
THP1: Human myeloid leukemia monocytes
HEK293T: Human embryonic kidney cells
TRAF6: TNF receptor associated factor 6
STAT1: Signal transducer and activator of transcription 1
