# Supplementary material for: MicroRNA-146a-5p-modified human umbilical cord mesenchymal stem cells enhance protection against diabetic nephropathy in rats through facilitating M2 macrophage polarization
Source: Stem Cell Res Ther. 2022 Apr 27;13:171. doi: 10.1186/s13287-022-02855-7 (PMC9044847; doi:10.1186/s13287-022-02855-7)
Supplement: Supplementary file 1 — Additional file 1: Fig. S1. UC-MSC-derived miR-146a-5p targets TRAF6 and facilitates M2 macrophage polarization in THP1. Fig. S2. TRAF6 is required for UC-MSCs-derived miR-146a-5p-mediated M2 macrophage polarization in THP1. Fig. S3. miR-146a-5p modification in UC-MSCs enhanced the efficacy renal pathological improvement. Table S1. Primers for qRT-PCR. Table S2. The sequence of miRNAs. Table S3. The target sequence of siRNAs [file 13287_2022_2855_MOESM1_ESM.docx]

This article contains the following supplemental material

Fig. S1 UC-MSC-derived miR-146a-5p targets TRAF6 and facilitates M2 macrophage polarization in THP1

Fig. S2 TRAF6 is required for UC-MSCs-derived miR-146a-5p-mediated M2 macrophage polarization in THP1

Fig. S3 miR-146a-5p modification in UC-MSCs enhanced the efficacy renal pathological improvement

Table. 1 Primers for qRT-PCR

Table. 2 The sequence of miRNAs

Table. 3 The target sequence of siRNAs

**
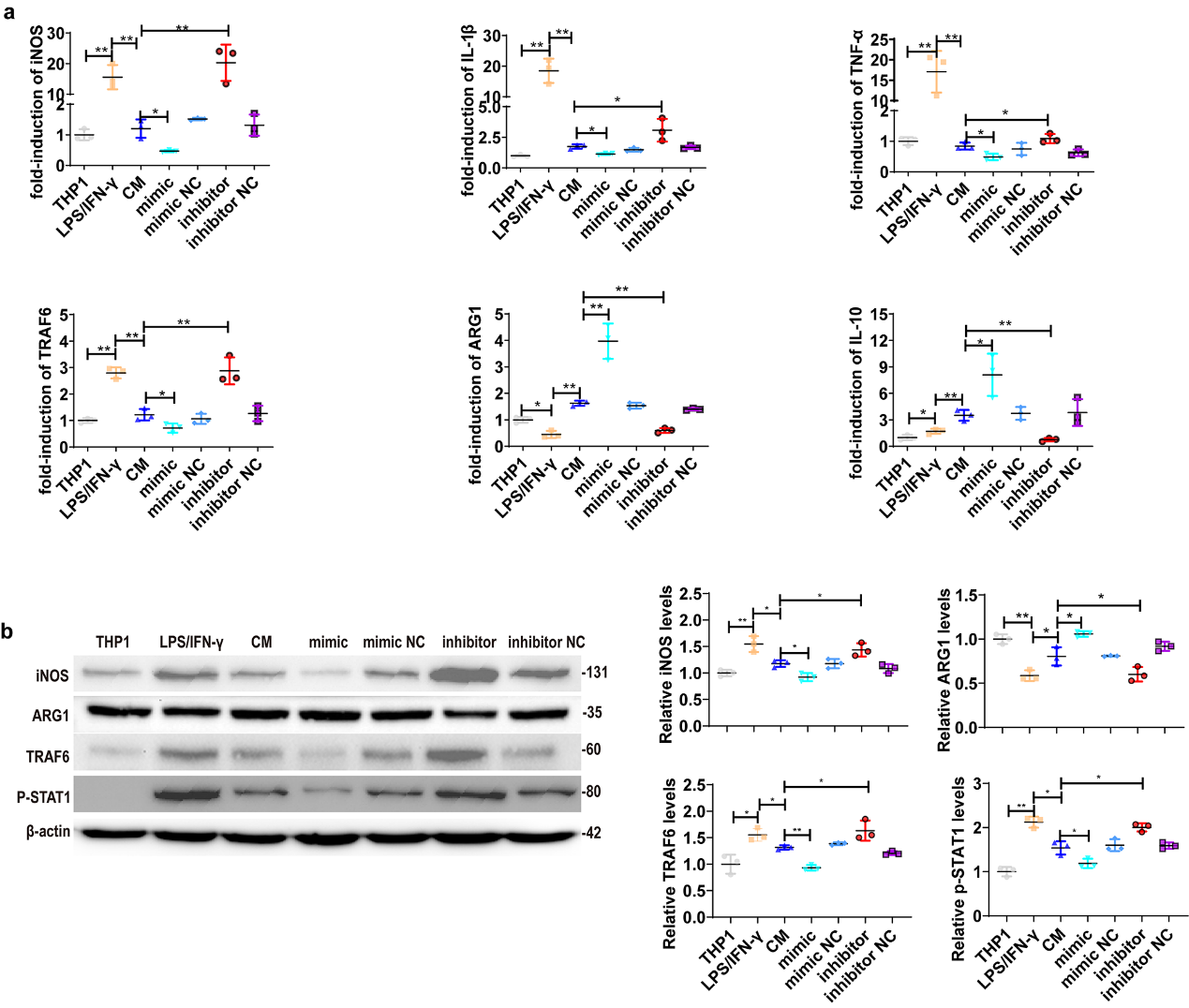
 Supplementary Fig.1 UC-MSC-derived miR-146a-5p targets TRAF6 and facilitates M2 macrophage polarization in THP1.** The miR-146a-5p mimic, miR-146a-5p inhibitor were transfected into UC-MSCs, and the CM was collected to treat THP1. **a** Relative mRNA expression of inflammatory cytokines and M1/M2 macrophage markers including iNOS, MCP1, IL-1β, IL-6, TNF-α, TRAF6, ARG1, and IL-10 in the control THP1, LPS/IFN-γ-stimulated THP1 and LPS/IFN-γ-stimulated THP1 treated with the indicated CM. **b** Relative protein expression and semi-quantitative analysis of iNOS, ARG1, TRAF6, and p-STAT1 in THP1. Data presented as mean ± SD in each group. Results in vitro are representative of three independent experiments. * P < 0.05, ** P < 0.01.

**
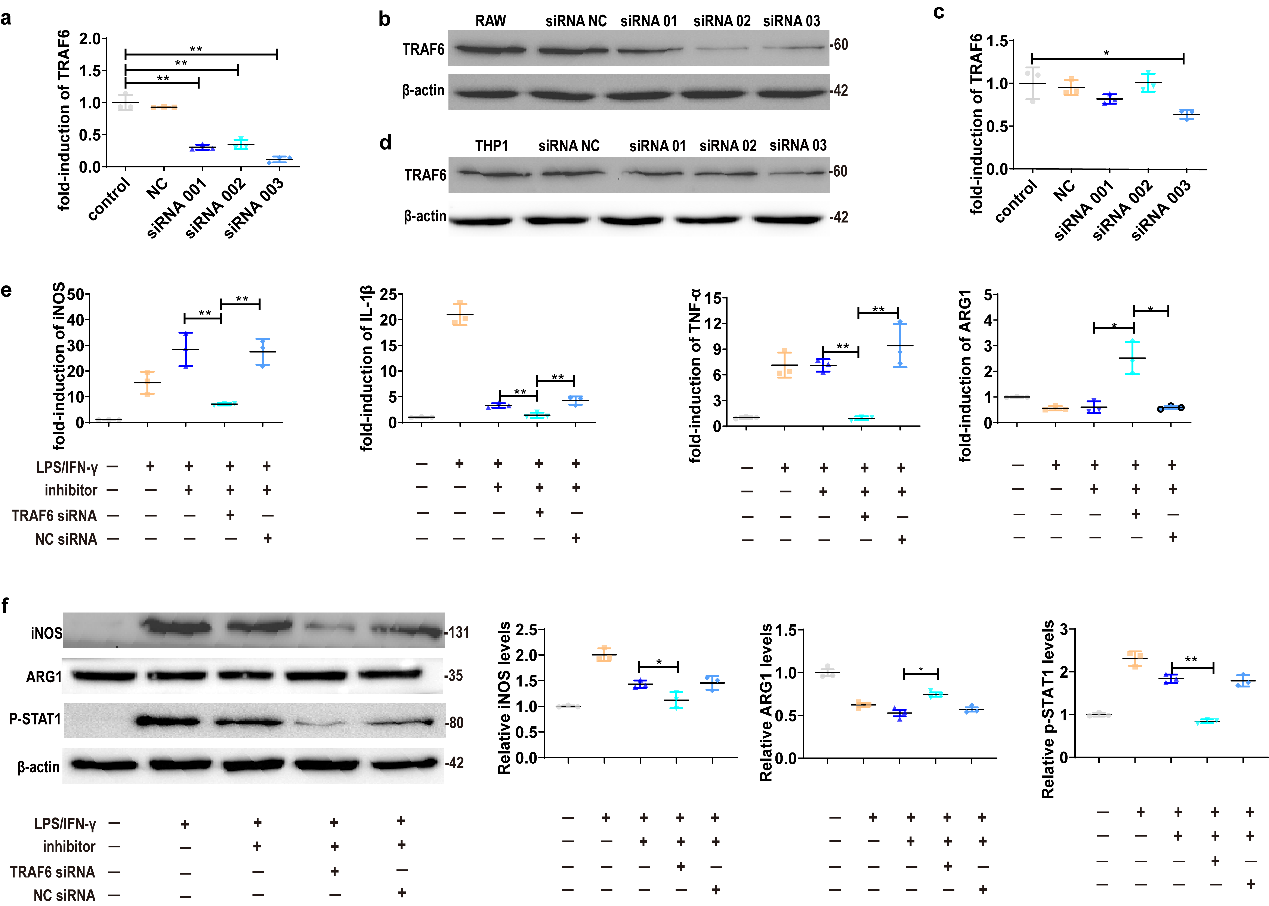
 Supplementary Fig.2 TRAF6 is required for UC-MSCs-derived miR-146a-5p-mediated M2 macrophage polarization in THP1.** THP1 cells were transfected with TRAF6 siRNA or negative control (NC) siRNA, and then treated with CM derived from miR-146a-5p inhibitor transfected-UC-MSCs. The mRNA level **a** and protein level **b** of TRAF6 in RAW264.7 after transfection with TRAF6 siRNA. The mRNA level **c** and protein level **d** of TRAF6 in THP1 after transfection with TRAF6 siRNA. **e** Relative mRNA expression of inflammatory cytokines and M1/M2 macrophage markers including iNOS, IL-1β, TNF-α, and ARG1 in THP1. **f** Relative protein expression and semi-quantitative analysis of INOS, ARG1, and p-STAT1 in THP1. Data presented as mean ± SD in each group. Results in vitro are representative of three independent experiments. * P < 0.05, ** P < 0.01.


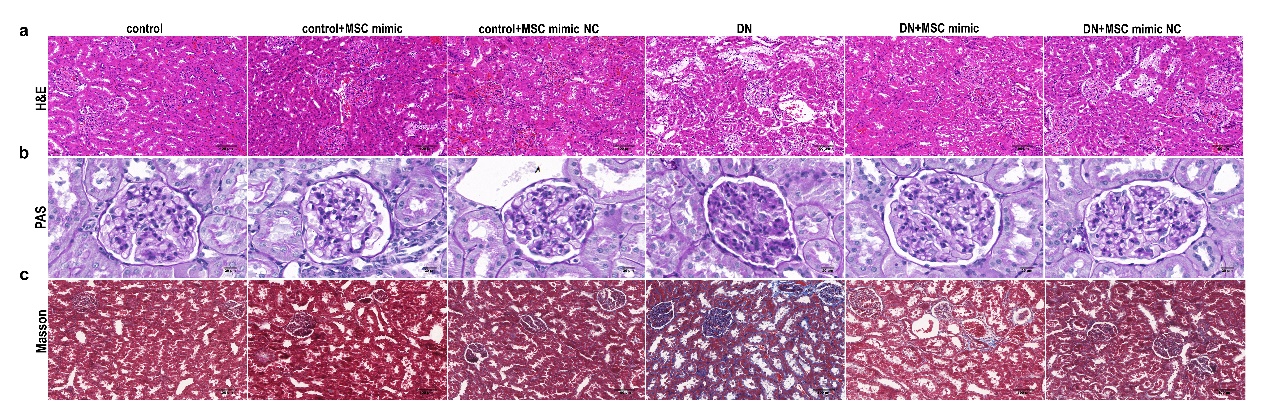


**Supplementary Fig.3 miR-146a-5p modification in UC-MSCs enhance the efficacy renal pathological improvement. a** H&E staining in the kidneys to observe the tubular dilatation and massive accumulation of inflammatory cells in the interstitial area. **b** PAS staining in the kidneys to observe the renal glomerular hypertrophy and collective apoptosis in tubular units. **c** Masson staining in the kidneys to observe the renal interstitial fibrosis, n=3 rats/group, Scale bar: 100 µm in H&E and Masson, Scale bar: 20 µm in PAS.

| Table. 1 Primers for qRT-PCR | |
| --- | --- |
| gene | sequence (5’-3’) |
| r GAPDH-F | TGATTTTGGAGGGATCTCGC |
| r GAPDH-R | ACGGATTTGGTCGTATTGGG |
| r IL-1β-F | CGACAGTGAGGAGAATGACC |
| r IL-1β-R | ACCACTTGTTGGCTTATGTT |
| r IL-6-F | CGGAGAGGAGACTTCACAGA |
| r IL-6-R | GGAGAGCATTGGAAGTTGGG |
| r TNF-α-F | TGCCTCAGCCTCTTCTCATT |
| r TNF-α-R | GTGGGTGAGGAGCACGTAGT |
| r IL-10-F | GTCCTTTCACTTGCCCTCATC |
| r IL-10-R | CAAACTGGTCACAGCTTTCGA |
| r iNOS-F | CTCACTGTGGCTGTGGTCACCTA |
| r iNOS-R | GGGTCTTCGGGCTTCAGGTTA |
| r ARG1-F | CGGCTTGCGAGATGTGG |
| r ARG1-R | TAGCCGGGGTGAATACTGG |
| m GAPDH-F | TGTGATGGGTGTGAACCACG |
| m GAPDH-R | CAGTGAGCTTCCCGTTCACC |
| m iNOS-F | CAGCTGGGCTGTACAAACCTT |
| m iNOS-R | CATTGGAAGTGAAGCGTTTCG |
| m MCP1-F | CTCACCTGCTGCTACTCATTC |
| m MCP1-R | TTACGGCTCAACTTCACATTCA |
| m IL-1β-F | ACGGACCCCAAAAGATGAAG |
| m IL-1β-R | CACGGGAAAGACACAGGTAG |
| m IL-6 -F | TGATGGATGCTACCAAACTGGA |
| m IL-6 -R | TGTGACTCCAGCTTATCTCTTGG |
| m TNF-α-F | CTTCTGTCTACTGAACTTCGGG |
| m TNF-α-R | TGATCTGAGTGTGAGGGTCTG |
| m TRAF6-F | CCTGACGGTAAAGTGCCCAA |
| m TRAF6-R | ACCTGGCACTTCTGGAAAGG |
| m ARG1-F | GATTATCGGAGCGCCTTTCT |
| m ARG1-R | CCACACTGACTCTTCCATTCTT |
| m IL-10 -F | GCCAGAGCCACATGCTCCTA |
| m IL-10 -R | GATAAGGCTTGGCAACCCAAGTAA |
| h GAPDH-F | CAAGTGAAATGATGGCTTATTAC |
| h GAPDH-R | CTTTCAACACGCAGGACAGGT |
| h iNOS-F | AGCCCTTTACTTGACCTCCTAA |
| h iNOS-R | CAAGTTCCATCTTTCACCCAC |
| h MCP1-F | GCAGCAAGTGTCCCAAAGAA |
| h MCP1-R | CTGGGGAAAGCTAGGGGAAA |
| h IL-1β-F | AGCCACATCGATCAGACACC |
| h IL-1β-R | GTACTCAGCGCCAGCTCG |
| h IL-6-F | TAATGGGCATTCCTTCTTCT |
| h IL-6-R | TGTCCTAACGCTCATACTTTT |
| h TNF-α-F | GGTGCCTATGTCTCAGCCTCTT |
| h TNF-α-R | GCCATAGAACTGATGAGAGGGAG |
| h TRAF6-F | TTTGGTCTTATGGATTGTCCCC |
| h TRAF6-R | GATTGATGCAGCACAGTTGTC |
| h ARG1-F | CTGTGGGAAAAGCAAGCGAG |
| h ARG1-R | CATGGCCAGAGATGCTTCCA |
| h IL-10-F | GAGGTGATGCCCCAAGCTG |
| h IL-10-R | CACGGCCTTGCTCTTGTTTT |

r-rat m-mouse h-human

| Table. 2 The sequence of miRNAs | |
| --- | --- |
| Name | sequence (5’-3’) |
| miR-146a-5p mimic sense | UGAGAA CUGAAUUCCAUGGGUU |
| miR-146a-5p mimic antisense | AACCC AUGGAAUUCAGUUCUCA |
| miR-146a-5p inhibitor sense | AACCCAUGGAAUUCAGUUCUCA |
| miR-146a-5p mimic NC sense | UUUGUACUACACAAAAGUACUG |
| miR-146a-5p mimic NC antisense | AAACAUGAUGUGUUUUCAUGAC |
| miR-146a-5p inhibitor NC sense | UUUGUACUACACAAAAGUACUG |

| Table. 3 The target sequence of siRNAs | |
| --- | --- |
| Name | target sequence (5’-3’) |
| si-h-TRAF6_001 | GTAGCGCTGTAACAAAAGA |
| si-h-TRAF6_001 | AGGGTCGCCTTGTAAGACA |
| si-h-TRAF6_001 | CATGCACATTCAGTACTTT |
| si-m-TRAF6_001 | GCCTGCATCATCAAATCCA |
| si-m-TRAF6_002 | TAAGCCAACCAGTTACTT |
| si-m-TRAF6_003 | CATTAAGGATGATACATTA |

h-human m-mouse
